# A Fiber-Optic Gas Sensor and Method for the Measurement of Refractive Index Dispersion in NIR

**DOI:** 10.3390/s20133717

**Published:** 2020-07-02

**Authors:** Matej Njegovec, Denis Donlagic

**Affiliations:** Laboratory for Electro Optics and Sensor Systems, Faculty of Electrical Engineering and Computer Science, University of Maribor, Koroska cesta 46, 2000 Maribor, Slovenia; denis.donlagic@um.si

**Keywords:** gas sensor, anomalous dispersion, gas refractive index, Fabry-Perot gas sensor, methane sensing

## Abstract

This paper presents a method for gas concentration determination based on the measurement of the refractive index dispersion of a gas near the gas resonance in the near-infrared region (NIR). The gas refractive index dispersion line shape is reconstructed from the variation in the spectral interference fringes’ periods, which are generated by a low-finesse Fabry-Perot interferometer during the DFB diode’s linear-over-time optical frequency sweep around the gas resonance frequency. The entire sensing system was modeled and then verified experimentally, for an example of a low concentration methane-air mixture. We demonstrate experimentally a refractive index dispersion measurement resolution of 2 × 10^−9^ refractive index units (RIU), which corresponds to a change in methane concentration in air of 0.04 vol% at the resonant frequency of 181.285 THz (1653.7 nm). The experimental and modeling results show an excellent agreement. The presented system utilizes a very simple optical design and has good potential for the realization of cost-efficient gas sensors that can be operated remotely through standard telecom optical fibers.

## 1. Introduction

Gas sensing using optical fiber sensors is becoming increasingly important in many areas of the industry due to the possibility for remote sensor location, high sensitivity, intrinsic explosion-safety, electromagnetic immunity and compatibility with harsh environments. The broad availability of laser diodes for the near-infrared region (NIR) allows for the observation of specific gas resonance lines [1] while preserving compatibility with optical fibers [2]. Laser diodes can be tuned rapidly, or modulated around a specific absorption peak [3], while information about gas concentration is extracted from the sensor response. The sensitivity of such sensors depends primarily on the optical path length in the gaseous medium and the modulation/demodulation technique. Consequently, gas cells often include multipass designs [3,4,5,6,7], or resonators with a large distance between mirrors [8]. Long gas or multipass gas cell designs, however, often increase the complexity of the optical setup and its alignment, which increase the size and the cost of the sensing system. The latter frequently limits the use of these approaches in industrial and similar applications.

Many methods for optical measurement of gas concentration based on absorption spectroscopy were investigated in the past [9,10], although, only limited work has been performed in gas sensing that utilizes observation of resonant refractive index change, e.g., the appearance of anomalous dispersion of refractive index around the gas resonance frequency. Reasons for limited interest in dispersion-based gas sensing can likely be traced to the need for very high-resolution measurement of a gas refractive index, which should be accomplished within a very narrow band of optical frequencies. Dispersion-based gas sensing was, thus, often considered as a low-sensitivity and complex approach [11]. 

One of the earliest works dedicated to measurements of dispersion around the resonance peak is based on a Hook method [12,13], which was later improved further in [11,14,15,16]. This method and its derivatives are based on the free-space interferometer, where the beam from the optical source is split into two beams, while directing one through a gaseous medium and the other through a vacuum. Beams are then combined to interfere, while the obtained interferogram is used to determine anomalous dispersion curves. In a more recent proposal [8], two 222 cm long high-finesse Fabry-Perot Interferometers were used to measure a gas dispersion in CO_2_. The system is based on a tunable CO_2_ laser (operating in mid-infrared range), with an optical frequency that overlaps with the sensing gas resonance. Laser optical frequency was modulated using external grating and PZT around the gas resonance, while, simultaneously, responses were acquired from both interferometers, and the fringe difference between sensing and reference Fabry-Perot interferometer (FPI) responses was then utilized to compute information about the dispersion of the refractive index. Measurement of refractive index was possible within a range of 2 × 10^−7^ RIU, although the system was not tested at different gas concentrations, and, at the same time, only pure gas was used for the experiment at a pressure of 5 torr. Furthermore, this system also operates in the mid-infrared (MIR) range, which makes it incompatible with low-loss telecom silica optical fibers (available MIR fibers have very high losses in comparison to silica fibers and are in most cases not suitable for optical signal transmission over even moderate distances), and requires the in-situ installation of the entire optical system, which would make the operation of this system limited in an out-of-the-laboratory environment. None of the above works was actually considered as a method for gas detection and concentration measurement but were rather used as a demonstration of the appearance of anomalous refractive index dispersion near resonance. In most of the above references, the authors concluded that the appearance of the anomalous dispersion is very weak.

In more recent works, the heterodyne phase-sensitive detection spectroscopy (HPSDS) [6,17] and chirped laser dispersion spectroscopy (CLaDS) [18,19,20] techniques were proposed to increase the sensitivity of dispersion measurement method to allow for measurements of low gas concentrations. In order to reconstruct anomalous dispersion curve of the gas refractive index with high sensitivity, such systems still require long (several meters) gas cell, a tunable laser source, an optical intensity modulation (HPSDS) or frequency modulation (CLaDS), and high-speed RF components. In case of HPSDS, optical frequency is swept linearly over time, while, simultaneously, optical power is sinusoidally modulated with high frequency. Gas dispersion is then extracted by using a heterodyne demodulation technique, which often requires the implementation of a lock-in amplifier. In case of CLaDS method, optical frequency is sinusoidally modulated while at same time optical frequency is linearly swept., Anomalous dispersion curve is reconstructed using FM demodulation techniques, and correlated to the gas concentration. Both methods utilized long sensing optical paths (100 m [6], 18 m [18] and 34 m [19]) in order to achieve high-sensitivity of gas detection. Furthermore, MIR Quantum cascade lasers (QCLs) were utilized in [6,19] due to considerable higher absorption peaks in MIR region. QCLs are, however, complex opto-electronic devices with limited potential for volume production [21], while use of MIR range prevents use of low-loss fibers for remote connection of sensor and signal interrogation system.

In this paper we propose a method and a design of NIR gas detection system for the measurement of refractive index dispersion of gas near its resonance, which relies on a small number of optical and optoelectronic components. The method relies on all-digital signal processing, which can be executed on a microcontroller or similar device rather than using complex modulation and/or demodulation techniques to reconstruct the refractive index dispersion curve. The system in the presented configuration achieves resolutions that can support a variety of field applications (for example explosion limit monitoring in methane exposed environments). The sensor is operated remotely through a standard telecom optical fiber, and is based on a simple, about 40 cm long, single-pass, low-finesse, Fabry-Perot gas cell, a thermally stabilized distributed feedback laser diode (DFB) derived from an L-band telecom transmitter, which has an emission wavelength near the selected gas resonance peak, a detector with amplifier, and a digital processing unit. The use of few and broadly available telecom components, undemanding sensing gas cell design and all-digital signal processing make the proposed system potentially low-cost, which represents one of the major challenges in the design and broader use of spectroscopic systems today. The principle of operation, detailed modeling of system response, processing algorithms, experimental setup, and experimental results are presented in detail within the article below.

## 2. Sensor Design and Operation

### 2.1. Principle of Operation

Gas detection and concentration measurement through measurement of dispersion require a sensitive and stable method for gas refractive index measurement over an optical frequency range near the examined gas resonance. The refractive index of a gas changes rapidly near the resonance, while these changes are usually small at concentrations of interest, especially when the sensed gas is present in a low concentration in a host atmosphere. For example, at atmospheric pressure, a few percent of the methane in air typically causes a change in RI of the gas mixture within the range of 10^−7^ RIU when changing the frequency of the probe light from non-resonance frequency to the resonance frequency at wavelengths near 1653 nm. This non-resonant to resonant and back to the non-resonant change in RI occurs within a wavelength span of about only 50 pm [3].

Traditional methods for reliable high-resolution RI measurements (especially those using optical fiber setups) usually utilize interferometers which are spectrally resolved [22,23]. However, the implementation of spectrally resolved methods for measurements of RI variation in gases near resonances proves to be challenging, due to the very narrow span of optical frequencies where a measurable change in the RI actually occurs.

**Figure 1:**
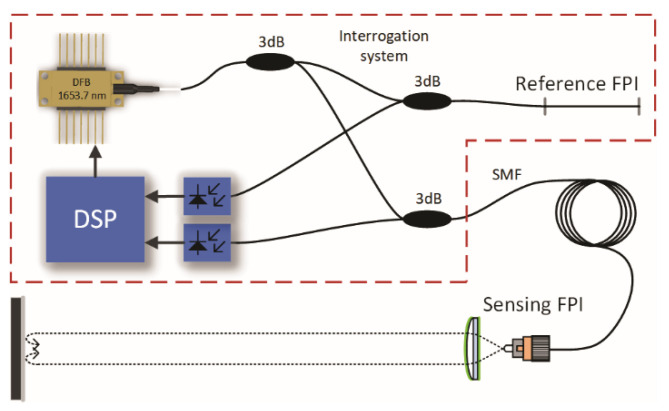
Basic system setup.

To provide a possibility for high-resolution spectral interrogation of gas RI within a narrow band of optical frequencies, we propose a system composed of a low-finesse gas sensing Fabry-Perot interferometer (FPI), a high-coherence laser source tunable over optical frequencies around the gas resonance, a reference interferometer and a signal acquisition and processing unit, as depicted in Figure 1.

The length of the low-finesse FPI is chosen in a way so that the free spectral range (FSR) of the interferometer is significantly narrower than the target gas spectral line. When an interferometer like this is filled with the target gas or gas mixture, and when the optical frequency is swept linearly over time across the gas resonance line, the light reflected from the interferometer forms a significant number of spectral interference fringes. In the absence of the target gas, these interference fringes are equally (fully periodically) spaced in the domain of the optical frequencies (and, consequently, in the time domain during the sweep), however, when the target gas is present within the interferometer, the spacing among individual fringes varies/changes due to the changes in RI near the gas resonance. Acquisition and proper signal processing of this fringe period variation thus provide an opportunity to perform sensitive and selective gas detection. Since the gas resonance is narrow, an appropriately current driven DFB laser diode, derived from telecom applications, can be used as a cost-efficient tunable laser source. Furthermore, a reference interferometer is added to provide means of compensation for the nonlinearity of the optical frequency sweep, which is required for proper detection of small variations in the spectral fringe period, as explained further below.

### 2.2. Low Finesse FP Interferometer Design

The proposed sensor was designed using an about 43 cm long Fabry-Perot interferometer with low finesse, which provides sinusoidal interference fringes in the domain of optical frequencies. An interferometer with a length of about 43 cm provides a free spectral range (FSR) of about 3.2 pm at 1653.7 nm (or 350 MHz in the frequency domain). When the DFB laser diode was swept for 250 pm or 16.5 GHz (a typical value that can be achieved by a simple diode drive current modulation) around the methane resonance line, the interferometer generated approximately 75 interference fringes, and about 30 of those fringes were spread over the gas resonance line.

**Figure 2:**
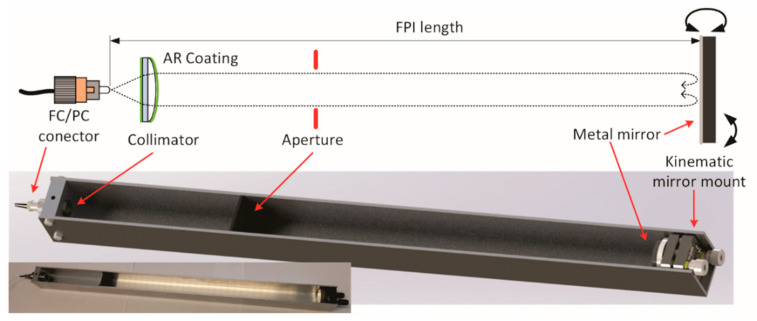
Low-finesse Fabry-Perot gas sensing interferometer.

In comparison to multipass or high finesse cells frequently used in gas sensing, a low finesse Fabry-Perot interferometer requires only a single roundtrip pass through the resonator, that can (and even should), exhibit reasonable high optical losses. This simplifies the optical design of the proposed sensor significantly in terms of used components, alignment precision and temperature/long term stability of the optical alignment. The experimental setup of the proposed FPI is shown in Figure 2, and consists of a lead-in single-mode fiber terminated with a fiber optic flat polished (FC/PC) connector, a single anti-reflective (AR)-coated collimation lens, a metallic (aluminum) mirror mounted on a miniature tilt stage, and a round aperture with a diameter of about 3 mm. 

All components were mounted into a simple aluminum U-shaped profile. A miniature tilt stage was used to align the direction of the back-reflected beam towards the collimating lens. The relatively long focal length of the collimating lens provides a beam diameter within the resonator in a millimeter range (about 3 mm in this particular case). Furthermore, we provided a slight and deliberate decollimation of the resonator beam, which increased the allowable angular alignment tolerances. This optically simplified setup is possible because only a few percent of the optical power at the output of the fiber needs to be back-reflected and recoupled into the fiber to form a low-finesse Fabry-Perot interferometer. A low-finesse Fabry-Perot interferometer is, thus, formed by a reflection from the FC/PC connector and the metallic mirror. The round aperture inserted in-between the collimator and mirror is used to prevent multiple light passes, and to mitigate unwanted reflections from the surrounding walls of the aluminum U-shaped profile. The aperture was positioned at a distance of about 100 mm from the collimating lens. The total length measured between both mirrors was about 43 cm.

## 3. Signal Interrogation and Modeling of the Demodulation Process

To describe the operation and predict the behavior of the proposed system, we obtained a model that describes the dispersion of the refractive index of a selected gas at different gas concentrations within a host atmosphere. A simplified model of an FP interferometer with a fringe period that is significantly narrower than the sensed gas resonance linewidth was then employed to model the interferometer’s responses to RI variation. The obtained results were used further to derive a signal processing algorithm, and later to compare the experimentally obtained data with the model.

### 3.1. Determination of the Refractive Index Variation Near the Sensed Gas Resonance

**Figure 3:**
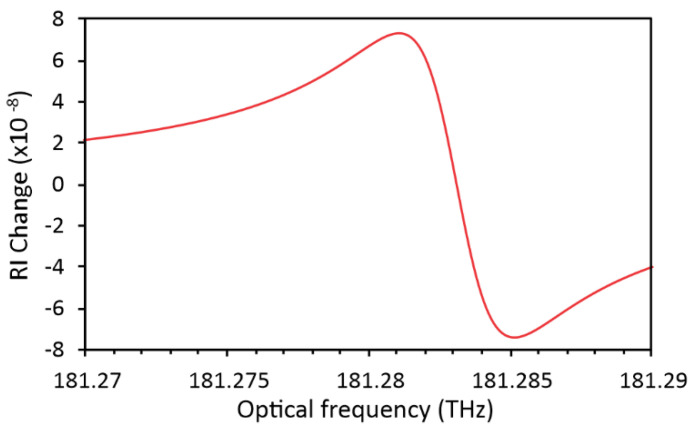
Simulated change of refractive index at 3 vol% methane concentration in the air.

The change in the refractive index around the resonance was obtained from the general equation for the dispersion of refractive index around the resonant frequency [24]:

(1) $$\Delta n\left(\omega \right)=\frac{1}{4\pi^{2}}\sum^{\text{​}}N_{i}CS_{i}\frac{\omega_{0i}-\omega }{{\left(\omega_{0i}-\omega \right)}^{2}+\gamma^{2}}$$

where *Ni* represents the density of the gas molecular species, *C* represents the sensed gas concentration in a host atmosphere, *S_i_* represents the line intensity of the ith line, *ω_0i_* represents the resonant frequency of the ith line, and *γ* represents the Lorentzian half-width at half maximum (HWHM). *γ* is sum of gas self-broadening and host (air) atmosphere broadening for selected air-gas mixture and is obtained from HITRAN database at selected resonant frequency. For an ideal gas at 296 °K and the pressure of 1 atm, *N_i_* is constant, and corresponds to 2.47 × 10^19^ molecules/cm^3^ [25]. In further simulations (if not stated otherwise), we assumed a 3 vol% concentration of methane gas (CH_4_) in an air mixture, at wavelengths around 1653.7 nm (181.285 THz). Values for *S_i_*, *ω_0i_*, and *γ* were obtained from the high-resolution transmission molecular absorption (HITRAN) database. Around the selected optical frequency, there are three very closely spaced spectral lines. Changes of RI of the gas were calculated according to Equation (1) for each spectral line, and then summed together to obtain the total RI change in the vicinity of 181.285 THz (1653.7 nm). The result of this calculation is presented in Figure 3.

A total expected RI change is, thus, in the order of 10^−7^ RIU in the case of 3 vol% of CH_4_ in the air. This change occurs within a frequency span of about 6 GHz (50 pm).

### 3.2. Interferometer Response and Operational Principle of the Algorithm for Reconstruction of Gas RI Dispersion Characteristics

The reflectance of a low-finesse FPI with a constant length *L*, but variable (i.e., frequency depended) RI in its optical path, can be described as:

(2) $$\frac{P_{R}}{P_{0}}=2R\left[1+\cos\left(\frac{4\pi Lf}{c}n\left(f\right)\right)\right]$$

where *P_R_*/*P*_0_ is relative reflectance, *R* reflectance of the interferometer’s mirrors, *L* the distance between mirrors, *f* the optical frequency, *c* the speed of light and *n*(*f*) the refractive index of the gas, which is frequency dependent.

From the above expression, local peak positions in the interferometer’s relative reflectance can be expressed as:

(3) $$m2\pi =\frac{4\pi Lf_{m}}{c}n_{m}$$

where *m* represents an integer fringe index in the optical frequency domain, *f_m_* is the optical frequency of the fringe/peak with the index *m*, and *n_m_* is the refractive index of the gas at optical frequency *f_m_*. The above expression can be rewritten in the form:

(4) $$f_{m}=\frac{cm}{2Ln_{m}}$$

The difference of optical frequency Δ*f_m_* among neighboring peaks (e.g., fringe periods) can be then expressed as:

(5) $$\begin{array}{ll} \Delta f_{m}=f_{m+1}-f_{m} & =\frac{c}{2L}\left(\frac{m+1}{n_{m+1}}-\frac{m}{n_{m}}\right)=\frac{c}{2L}\left(\frac{n_{m}+mn_{m}-mn_{m+1}}{n_{m}n_{m+1}}\right)\\ & \approx \frac{c}{2L}\frac{1}{n}+\frac{c}{2L}m\frac{\delta n}{n^{2}}=\Delta f+\delta \Delta f_{m} \end{array}$$

The approximation in the last line of the above expression is obtained by the assumption that *n_m_n_m+1_* = *n^2^*, *n_m+1_* = *n*, while using designation *δn_m_* = *n_m_* − *n_m+1_*.

The first term of the above expression can be considered constant, while the second term relates to the change in the refractive index *δn* between the neighboring resonant optical frequencies with the change in the spectral fringe period *δ*(Δ*f_m_*), i.e., if the refractive index is frequency independent (constant) then the second term becomes zero, and Equation (6) yields a well-known expression for the free spectral range of an FP interferometer Δ*f* = *c*/(2*nL*). However, in the case when the refractive index is frequency-dependent, the spectral fringe period becomes modulated in the optical frequency domain, which is described by the second term of the above expression. The second term of the above expression can be further rewritten in the form:

(6) $${\delta n}_{m}=\frac{2Ln^{2}}{cm}\delta \Delta f_{m}\approx \frac{n}{f_{m}}\delta \Delta f_{m}$$

Since the variation of a spectral fringe period *δ*(Δ*f_m_*) can be measured with high resolution and directly by performing a linear optical frequency sweep, measurement of *δ*(Δ*f_m_*) provides an opportunity for reconstruction of the absolute value of the refractive index as a function of optical frequency.

To obtain the refractive index *n* at an arbitrary frequency *f_m_*, all changes/variations in the refractive index *δ*(Δ*f_m_*) between the fringe with index 0 and *m* = *m*(*f_m_*) shall be summed:

(7) $$n\left(f_{m}\right)=\frac{n}{f_{m}}\overset{m=m\left(f_{m}\right)}{\underset{m=0}{\sum }}\delta \Delta f_{m}=\frac{n}{f_{m}}\overset{m=2Lnf_{m}/c}{\underset{m=0}{\sum }}\delta \Delta f_{m}$$

Since the changes of interest in RI occur only near the gas resonance (far from the resonance, the refractive index is constant and *δ*Δ*f_m_* = 0), summing limits can be chosen in a way to take into account the optical frequency region where the refractive index is indeed non-constant, i.e., around the gas resonance line. Thus, the initial fringe index *m_start_* can be chosen in a way that corresponds to the optical frequency, which is somewhat higher than the gas resonance (typically, a few widths of the gas spectral line higher than the resonance line). It is, therefore, sufficient to choose the initial frequency of the laser sweep range in a way so as to be a few gas spectral linewidths higher than the observed gas resonance optical frequency. Furthermore, since the addition is performed only around the gas resonance, it does not take into account contributions of the other resonances that are far away from the selected resonance but contribute to the absolute refractive index. Thus, to obtain the absolute refractive index, the initial (out-of-the-resonance) refractive index n_0_, shall be added to the sum of Equation (7). Consequently, by assuming *m_start_* = *2L*/*cnf_start_*, the above expression (Equation (7)) can be rewritten as:

(8) $$n\left(f_{m}\right)=n_{o}+\frac{n}{f_{m}}\overset{m=2Lnf_{m}/c}{\underset{m=2Lnf_{start}/c}{\sum }}\delta \Delta f_{m}$$

**Figure 4:**
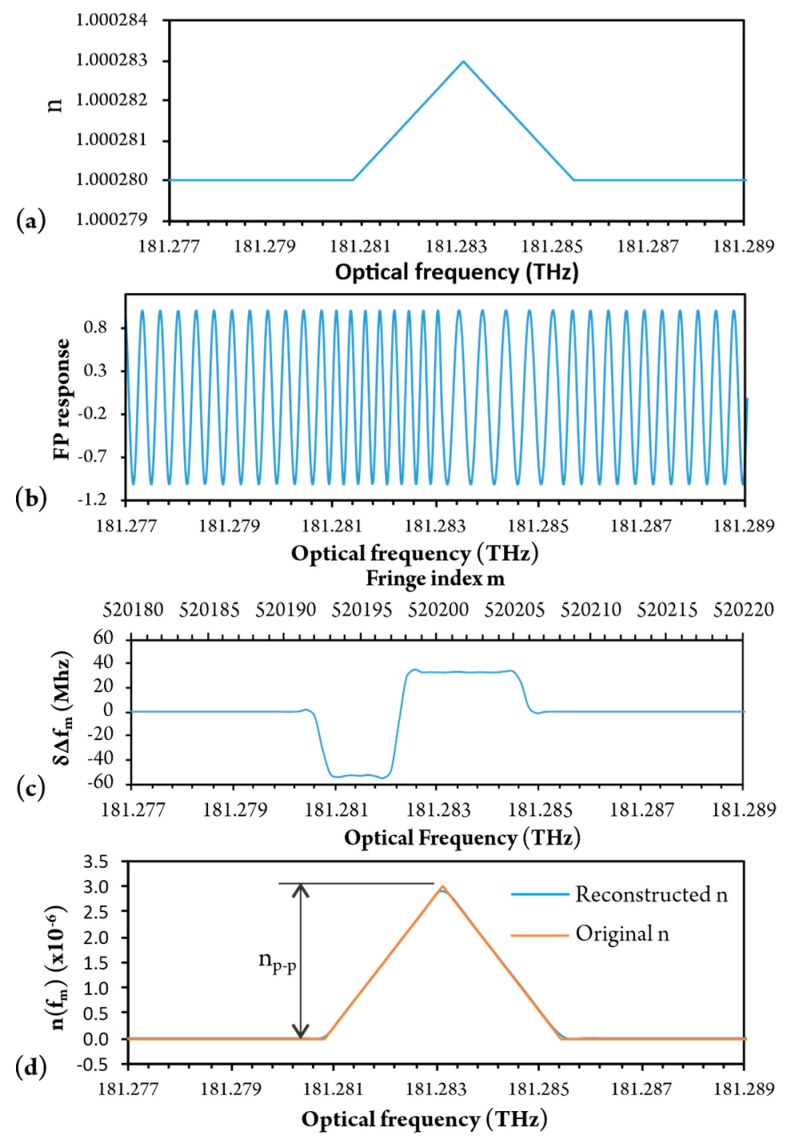
(**a**) Change of refractive index; (**b**) Response of FPI; (**c**) Fringe period variation; (**d**) Reconstructed refractive index variation.

To demonstrate the above-proposed RI demodulation method, we modeled the response of a 50 cm long FPI during the linear optical frequency sweep. We assumed that the interferometer was filled with a mixture of air (*n* = 1.00028) and a hypothetical gas that would cause a triangular change of RI at the resonant frequency with the amplitude of 3 × 10^−6^ as presented in Figure 4a (we deliberately chose an about 20 times larger change in RI than is expected in the case of 3 vol% air-CH4 mixture, in order to present the change in the fringe period better within simulated figures).

The simulated response of FPI is presented in Figure 4b, and shows clearly changes in optical frequency fringe periods in the regions where RI varies, i.e., the fringe period decreases in a region where Refractive Index increases, and the fringe period increases in a region where RI decreases. Thus, by measuring the difference in optical frequency between individual peaks, we obtain the fringe period as a function of fringe index m. To obtain a variation of the fringe period which is caused by RI variation, we subtracted the initial (non-resonant) fringe period (i.e., the fringe far away from the resonance), from the fringe period obtained by neighboring peaks (Figure 4c). This corresponds to the calculation of *δ*Δ*f_m_* as a function of fringe index *m*, as discussed in the initial part of this section. This change in the fringe period is summed according to Equation (8), in order to obtain RI versus optical frequency, as presented in Figure 4d. Figure 4d resembles the shape and amplitude of the initial RI curve clearly over the frequency range of interest. The maximal change/variation in the refractive index, which is proportional to the observed gas concentration, can thus be determined by measuring the difference between the minimum and the maximum value *n_p-p_* in the curve, as indicated in Figure 4d.

### 3.3. Implementation of Interrogation and Demodulation Algorithm

**Figure 5:**
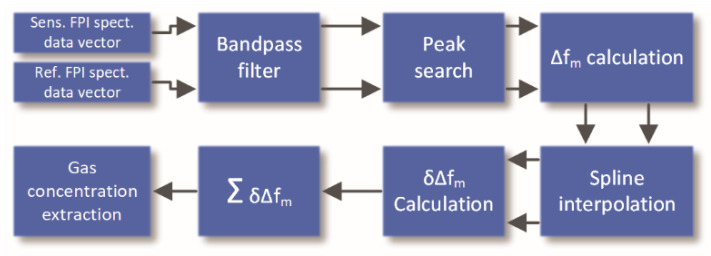
A basic algorithm to extract changes in refractive index.

To extract gas concentration from the FPI response we developed a measurement process based on the approach described in the previous section. The flow chart of the entire algorithm is presented in Figure 5.

To mitigate the negative effects of frequency sweep nonlinearity, we introduced a reference interferometer with the same optical path length as the sensing interferometer.

The measurement process begins with a linear sweeping of the laser source optical frequency around the gas resonant frequency. Simultaneously with the linear frequency sweep, the digital processing unit acquires responses from the sensing and the reference FPIs and submits the acquired data to the signal processing algorithm. The algorithm firstly filters both recorded responses by using a 6th order digital band pass filter. The central frequency of the band-pass filter matches the frequency of the generated interference fringes. The band-pass filter eliminates possible higher harmonics in recorded signals, which may be caused by multiple reflections within FP interferometers and improves the overall signal to noise ratio. At the same time, the offset (DC (direct current) signal) is removed from the acquired signal.

Peak positions in the optical frequency domain are then extracted by a peak searching algorithm (the time axis is converted to an optical frequency from the laser diode initial optical frequency and optical frequency sweep slope, which was acquired during the laser diode calibration process). The period of each fringe in the recorded data is further determined by subtracting the neighboring peak positions. Spline interpolation is then used to obtain continuous functions/relationships that relate to the variation in the fringe period frequency with optical frequency. There are two reasons for application of spline interpolation: firstly, the reference FPI is fiber-based, and is therefore sensitive to environmental temperature variations, meaning that the phase of acquired reference signal will vary randomly with the temperature and over time. This causes misalignment between the fringes of the sensing and the reference FPI in the optical frequency domain. Spline interpolation over both fringe period data sets allows for resampling of reference interferometer fringe period data, and their alignment with the sensing interferometer fringes in the optical frequency domain, which is required for the successful subtraction of both datasets, as described further below. Secondly, the observed peak positions are not ideally distributed, due to noise effects and errors in the peak search algorithm, therefore spline interpolation smoothens the fringe period data sets and decreases noise in processed data.

Computed and aligned (resampled) fringe periods of sensing and reference FPI are then subtracted, which compensates for possible nonlinearities in frequency sweep of the laser source and provides information on the variation in the fringe period (*δ*Δ*f_m_*), which originates only from the RI variation (and not from the nonlinearity of the laser frequency sweep). Subtraction of a fringe period far from the resonant optical frequency (described in the previous section) to obtain *δ*Δ*f_m_* is, thus, not needed. Summing the fringe period variations over the available data set finally results in refractive index values as a function of optical frequency. The amplitude of RI variation around the gas resonance (which is simply determined as a difference in local minimum and maximum within the curve) is then used as final output data that are correlated to a target gas concentration.

### 3.4. Simulation of the Entire Sensing System Based on Numeric Models

For a better representation of the above demodulation process, we performed a complete simulation of the demodulation algorithm presented in the previous section using the FPI model and RI data obtained by the gas model presented in Section 3.1. The simulation assumes a methane-air mixture with methane concentration within a range of 3 vol%. Within this simulation, we also assumed realistic data used later in the experimentally built sensor (43 cm long sensing FP, resonant peak at 181.2836 THz (1653.7 nm), and 16.443 GHz (250 pm) wide optical frequency sweep).

**Figure 6:**
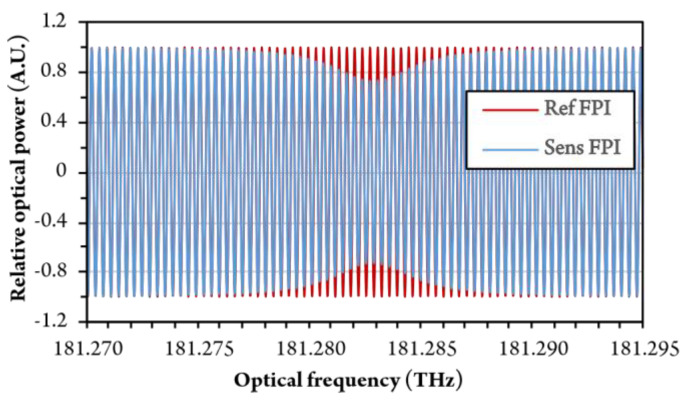
Simulated response of sensing and reference FPI at 3 vol% concentration of methane.

Refractive Index data for a given wavelength range and for a 3 vol% air-methane mixture was first calculated using Equation (1) and inserted into a low-finesse model of an FP interferometer (Equation (2)). To obtain fully realistic conditions, the model was also updated with absorption data obtained by using an online simulation tool SpectraPlot [26]. Simulated responses of the sensing and the reference FPI are presented in Figure 6. The limited drop in amplitude of the sensing FPI response at the resonance wavelengths is, thus, absorption related. Since the change in refractive index is small (within the 10^−7^ RIU range), the change in the fringe period around resonance frequencies is not clearly visible from the plot in the Figure 6.

**Figure 7:**
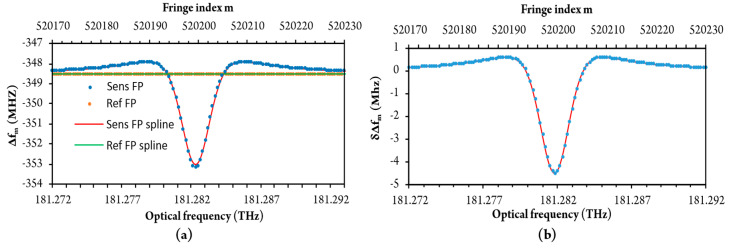
(**a**) The fringe period of sensing and reference FPI around resonant frequencies (fringe indexes) and (**b**) Fringe period variation (δΔf_m_) between sensing and reference FPI.

These data were then submitted for processing by the algorithm presented in the previous section. Figure 7 shows the calculated fringe period versus fringe indexes for both FPIs as it was obtained by the peak positions search algorithm. Spline interpolation, which was used to smooth out and align these data, is also shown in Figure 7a. Figure 7b shows the difference between sensing and reference fringe periods versus fringe indexes (after sensing and reference fringe periods were subtracted). This difference represents a fringe period variation that was caused purely by RI variation.

**Figure 8:**
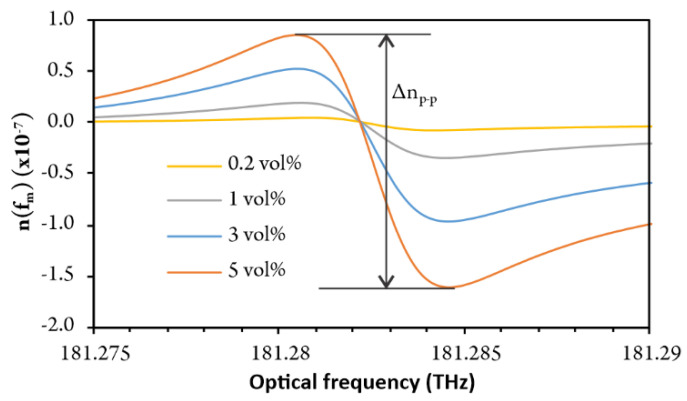
Reconstructed change in refractive Index at different gas concentrations (0.2, 1, 3 and 5 vol%) around resonant frequencies.

The summing of the curve presented in Figure 8 (using Equation (8)) finally yielded a curve that resembles the initial refractive index change. This curve was calculated for different volumetric gas concentrations. The curve corresponding to 3 vol% concentration clearly and closely resembles the original RI dispersion curve (Figure 3), which confirms the functionality and accuracy of the proposed demodulation approach. Finally, minimum and maximum were searched within the given frequency band, and the difference in amplitude of both was calculated to yield a parameter (Δ*n_p-p_*) proportional to the gas concentration.

## 4. Experimental Setup

**Figure 9:**
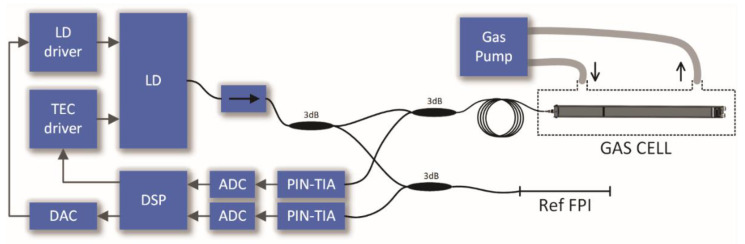
Experimental setup.

To evaluate the proposed approach experimentally, we built the experimental setup shown in Figure 9. The interrogation system consists of a fiber-coupled DFB diode SBF-D653S2-110P8 (Wuhan Shengshi Optical Technology Co., Wuhan, China) with linewidth of lower than 10 MHz (0.1 pm), integrated into a butterfly case with a thermo-electric cooler (TEC), sensing and reference interferometers, two telecom detectors with transimpedance stages, and a digital signal processing unit. The DFB diode was driven by a programmable current generator (consisting of a 16-bit digital to analog converter and analog driver), while the TEC driver provided programmable temperature control of the DFB diode.

The temperature of the diode was stabilized and adjusted to provide the initial emission wavelength of the DFB diode at 1653.65 nm (181.2913 THz). Optical frequency sweep of the laser diode was then achieved by the continuous sweeping of the diode’s drive current between about 23 and 48 mA, which provided the emission optical frequency sweep between 181.2913 and 181.2749 THz (1653.65 and 1653.8 nm in the wavelength domain). Sweeps were performed at a repetition rate of 46 Hz, while the sweep duration was 1.4 ms and was linearized in the optical frequency domain, as explained in detail below.

The DFB laser diode was connected to the gas sensing system through a dual-stage optical isolator to isolate the diode fully from the relatively significant back-reflected optical signals. An optical splitter divided the emitted optical power into two independent channels, one to the gas sensing FP Interferometer and the other to the reference FP interferometer. The reference interferometer was made out of a straight section of the single-mode fiber and was defined by an about 4% reflectance in-fiber mirror and flat cleaved fiber end, thus yielding in a low finesse FPI. The in-fiber mirror was produced by the process described in [20]. Lengths of reference and gas sensing interferometer were matched roughly during the cleaving process of the fiber that formed the reference interferometer, and then finally by fine-tuning the position of the mirror (using kinematic mirror mount) in the gas sensing interferometer. During matching process, responses of both sensors were monitored. When lengths of both interferometers were matched the same number of fringes was generated. The reference interferometer, which was physically about 29.3 cm long (corresponding to an FP interferometer optical length of about 43 cm), was further packed into a compact expanded polystyrene box to limit temperature gradients/fluctuations of the reference interferometer.

The gas sensing interferometer assembly was inserted into a compact tubular gas chamber, with an inlet and an outlet that were connected to a gas pump, which assured circulation and even distribution of the gas within the chamber. Gas piping included also input and output valves, which allowed for the controlled insertion of the selected gas into the gas chamber, and also venting of the chamber with the surrounding air (removal of sensing gas).

Optical signals reflected from the gas sensing interferometer and reference FP interferometer were acquired by a standard fiber coupled telecommunication InGaAs photodetectors and converted into voltage signals by a multichannel variable gain transimpedance amplifier (TIA) AS89000 (AMS, Premstaetten, Austria). The transimpedance amplifier gain was set to 1 MΩ, while the bandwidth was greater than 110 kHz. Electrical signals from transimpedance amplifier were further digitized by the 12-bit analog to digital converter, which was incorporated in the digital signal processor (DSP), while removing the DC component prior to digitalization. Signals were digitized synchronically and over the duration that corresponded to the laser diode drive current sweep. This provided a set of data, which represented interferometers responses as a function of optical frequency. The obtained digitized signal vectors were then sent to a personal computer for further processing and presentation of the results using the same algorithm as presented in the previous section.

### Laser Diode Calibration

Since the proposed system relies on the detection of small variations in fringe periods, the optical frequency sweep shall be extensively linear, as any non-linearity in the optical frequency sweep results in an undesired fringe period modulation. Thus, special attention was devoted to the linearization of optical frequency sweep and mitigation of effects related to residual nonlinearities. This proved to have an important effect on the achievable final resolution (in spite of using reference interferometer).

The relation between the change in the laser diode drive current and laser diode emission optical frequency is only approximately linear. It is, thus, necessary to find and correct the laser diode’s drive current shape in a way to obtain a substantially linear optical frequency sweep. This was accomplished by an appropriate iterative calibration process using an existing experimental setup. In the process of calibration, the laser diode temperature was first stabilized to the temperature that provided an emission optical frequency near the selected absorption peak. Then, the first current sweep cycle was executed by driving the laser diode using a linear current ramp, while recording the response from the reference FP interferometer. By knowing the optical path length of the reference interferometer and by measuring the fringe period between all recorded fringes, it is possible to reconstruct the emitted optical frequency as a function of sweep time. The measured optical frequency sweep was then subtracted from the target (linear) optical frequency sweep to get an error function, which was then multiplied with the selected gain factor and subtracted by the previous current ramp to get values for a new current ramp curve.

**Figure 10:**
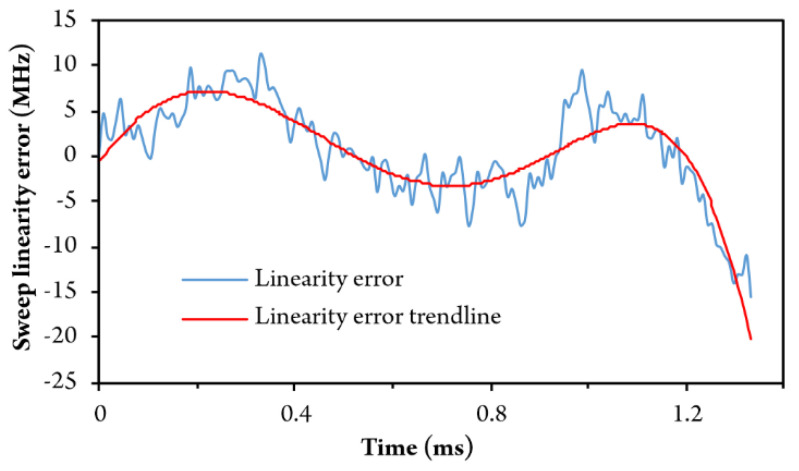
Optical frequency sweep non-linearity obtained after calibration process.

The entire process was repeated and, after several iterations, frequency sweep nonlinearity dropped below ±5 MHz, as shown in Figure 10, which corresponds to a non-linearity of ±0.03% at 16.4 GHz optical frequency sweep range (250 pm in the wavelength domain). Utilized calibration method is simple, fast and can be performed fully automatically.

## 5. Experimental Results

The proposed system was evaluated experimentally for sensing of the methane gas in air, as already described in previous sections. To demonstrate the operation of the proposed experimental system, we filled the experimental gas cell with ambient air. Then, we injected a small amount of methane into the gas cell to reach the desired methane-air concentrations. Methane was always inserted at room temperature and at normal atmospheric pressure (the pressure inside the gas cell was always equalized to environmental pressure after the gas was mixed).

**Figure 11:**
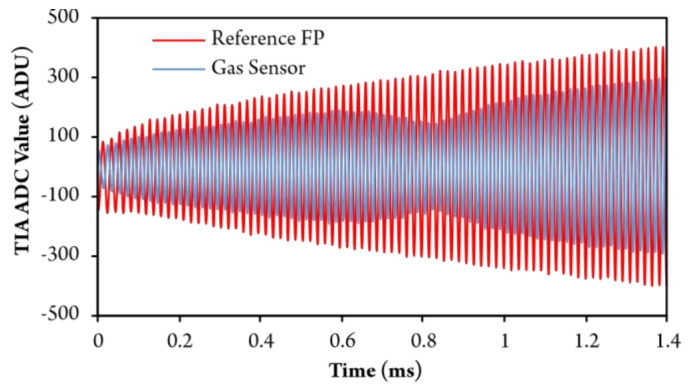
Digitized Response in analog-to-digital units (ADU) from the gas sensor and reference FP interferometer during the optical frequency sweep.

During testing, the laser diode optical frequency was always swept for 16.4 GHz (250 pm) within 1.4 ms. Recorded TIA digital responses from the gas sensor and reference FP Interferometer are, for example, shown in Figure 11.

**Figure 12:**
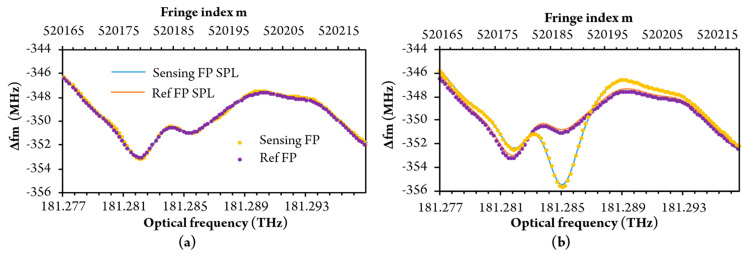
The fringe period of sensing and reference FP versus optical frequency (and fringe index) Without methane (**a**), and with 3 vol% methane (**b**).

Around 74 fringes were acquired within the selected optical sweep range (selective gas absorption is visible at the gas sensor in the middle of sweep range at higher methane concentrations). Acquired responses were preprocessed, firstly, by averaging of data obtained during four sweeps. These preprocessed data were then transferred to a PC for further processing by the algorithm described in Section 3.3. Fringe period versus fringe index (optical frequency) for pure air and 3 vol% methane-in-air mixture is shown in Figure 12.

**Figure 13:**
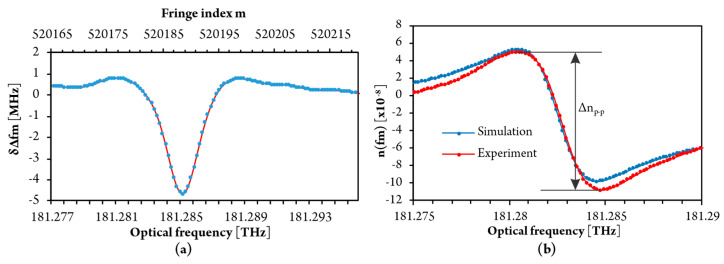
(**a**) Fringe period variation obtained by subtraction of the sensing and reference fringe period and (**b**) experimentally measured and simulated dispersion of Refractive Index.

Figure 12 shows that the experimentally obtained fringe period vs. fringe index deviates from simulation results (Figure 7a). The main cause for this deviation resides in the residual nonlinearity of the optical frequency sweep of the laser source. This is mitigated efficiently by subtraction of the sensor and reference interferometer fringe period versus fringe index data, as already explained in Section 3.4. and is shown in Figure 13a. The experimentally obtained data in Figure 13a concurs well with the simulated response shown in Figure 7b. The reconstructed refractive index frequency dependence obtained by the summing of *δ*Δ*f_m_* is shown in Figure 13b, which also presents a comparison between experimental and simulated results.

A good agreement between properly processed experimental data and the model is clearly visible. The gas concentration was then correlated to the amplitude of the difference between the peak and the valley of the curve shown in Figure 13b.

To establish a sensor’s static characteristics, i.e., the correlation between the amplitude of peak-to-valley difference Δ*n_p-p_* and actual methane concentration, we firstly evacuated the gas chamber fully using the vacuum pump and refilled it with the ambient air. Then, small, predetermined amounts of pure methane were injected repetitively into the chamber, to increase the methane concentration gradually in the desired concentration steps. Since the additional volume of introduced gas increases pressure within the gas chamber, it was also necessary to equalize pressure to atmospheric pressure prior to measurement (after the gas was mixed).

**Figure 14:**
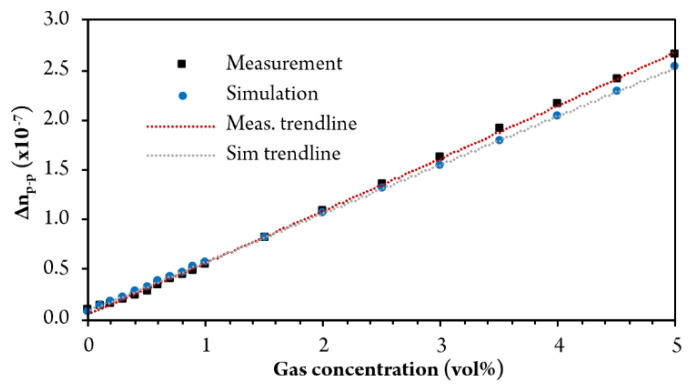
Static characteristics (RI change vs. gas concentration).

Figure 14 shows the sensor response (amplitude of peak-to-valley difference variation in the integrated fringe period curves) for methane concentration between 0 and 5%. Measurement of higher concentrations would also be possible (up to about 15% with the presented setup), although, the upper limit is limited mainly by gas absorption.

The acquired static characteristic is linear and fits well with the simulated response. From the static characteristics, we obtained the sensitivity of the sensor, which is defined as the ratio between the volumetric gas concentration and the change in the refractive index. The sensitivity for methane is thus 216 × 10^5^ vol%/RIU. A similar experiment was also performed to determine a system resolution. In this experiment, we injected small amounts of methane into the gas cylinder until the injection of the gas did not produce a clear change in the output signal that exceeded the system noise level.

**Figure 15:**
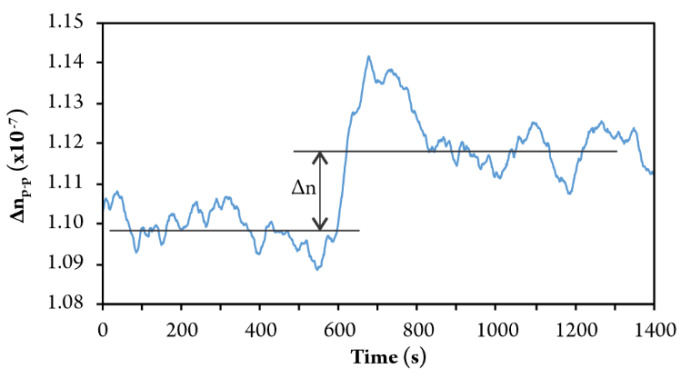
Injection of methane that increased Vol. concentration for 0.04%.

Figure 15 shows the response of the sensor during the injection of methane in the amount that increases the concentration of methane by 0.04 vol%. Thus, an increase in the concentration of 0.04 vol% clearly induced the output change that is larger than the sensor’s output noise, and the proposed system resolution can be estimated to be better than 0.04 vol% for the case of methane gas sensing.

These results were obtained using additional filtering of the output signal (a moving average of 20 samples), which provided a sample rate of 1 sps. It should, however, be stressed that a significant amount of time was lost for data transfer between the optoelectronic control unit and the PC. In addition, all PC algorithms were written in LabVIEW (NI, Austin, TX, USA), which has limited real-time processing capability. Implementation of embedded/on-board algorithms and the use of faster DSP platforms might, thus, further and significantly improve the sampling rate, and might thus further improve the signal to noise ratio of filtered signals and, consequently, system resolution.

## 6. Application to Other Gases 

**Figure 16:**
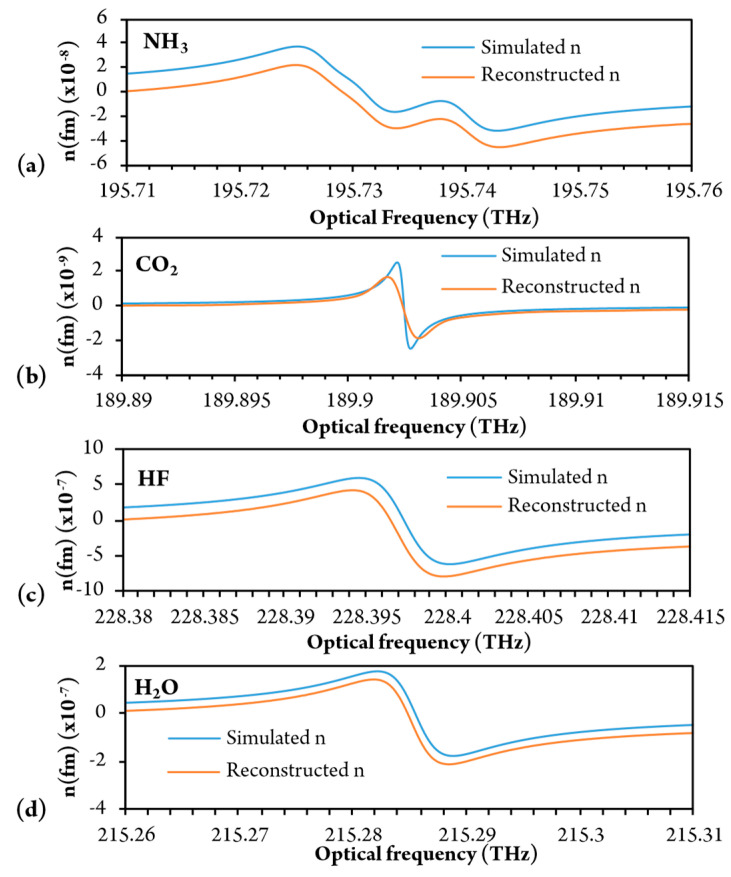
Simulated results for: (**a**) ammonia; (**b**) CO_2_; (**c**) water vapor using the same sensing interferometer.

In the preceding analysis, experimental and theoretical results were obtained only for methane gas. To analyze the sensing potential of the presented system further, we performed additional simulations for examples of different gases using the same FP model and algorithms. Ammonia, CO_2_, HF, and water were selected for simulation, and sensor sensitivity and resolution were correlated to the results obtained by the methane gas experimental setup (we assumed that the RI resolution would be the same for all gases). Simulated and reconstructed refractive index dispersion curves for each gas are presented in Figure 16 for the gas concentrations of 3 vol%.

A minor offset between initial and reconstructed signals is present because of the integration process, where information about the initial refractive index is lost. The estimated results are presented in Table 1.

The best dispersion sensitivity and resolution can likely be obtained for hydrofluoric acid and water, followed by methane and ammonia. Lower resolution and sensitivity could probably be achieved for carbon dioxide, due to the very weak spectral line intensity at the selected optical frequency. The performance of the CO_2_ sensing could, however, be improved by the increased optical path length of the FP Interferometer. By using a telecom-derived DFB laser diode with linewidths of about 10 MHz (maximum linewidth) we can estimate that maximum length of sensing FPI could be over 7 m long, which could further significantly increase sensitivity of the system. It is also worth mentioning that there are commercially available fiber-coupled laser diodes for all the above-presented gases and resonant optical frequencies. The presented system could, thus, be used for several gases that have suitable resonances in NIR and available laser diodes that fit into proper wavelength/frequency regions. Furthermore, proposed sensing method could be applied to quantum cascade laser (QCL) diodes as well (i.e., for MID range), as long as reference arm can be realized for frequency sweep nonlinearity compensation.

## 7. Conclusions

We demonstrate a sensor for the measurement of gas concentration, which utilizes the high-resolution measurement of reactive index variation near the gas resonance in the NIR region. The pro-posed sensor utilizes only a few electronic and optical components and an open path, low finesse Fabry-Perot interferometer with a length of about 40 cm. Utilization of a low finesse interferometer with a given length allows for a very simple and cost-effective opto-mechanical design. All optoelectronic components were borrowed or derived from telecom applications. The signal integration system and the sensor were connected through a standard single-mode optical fiber.

The system utilizes spectral interrogation, by performing a linear optical frequency sweep across the gas resonance in the NIR. An appropriate and straightforward signal-processing algorithm was developed that allowed for the extraction of gas RI variation near the resonance through observation of variation of the spectral interference fringe periods. The entire sensing system, gas resonance Refractive Index variation and signal processing algorithm, were modeled and then implemented experimentally. The experimental setup was adopted for sensing of low concentration of methane in the air. Experiments and modeling were in excellent agreement. The experimentally measured system resolution proved to be around 0.04 vol% when using methane gas resonance near 181.285 THz (1653.7 nm).

Modeling was also extended to other gases with similar resonances in the NIR region. Modeling results show that similar results could very likely be achieved experimentally with gases like ammonia, HF and water vapor (telecom derived DFB diodes for these gases are also commercially available). While the presented system in the presented form does not provide advancement in achievable gas sensing concentration resolution, when compared to several other spectroscopic systems, it offers the possibility for the design of a compact and cost-efficient sensor system that can utilize very basic opto-mechanical and efficient optoelectronic design, and can easily be connected remotely through the standard single-mode fiber. Furthermore, sensitivity could be further improved using Fabry-Perot resonators with longer optical path lengths (up to 14 m using present laser diode).

## Figures and Tables

**Table 1:** Estimated resolution assuming RI resolution of 2 × 10^−9^ RIU.

Gas	Peak to Peak Amplitude (at 3 vol%) [RIU]	Sensitivity [× 10^5^ vol% RIU^−1^]	Estimated Resolution [vol%]
CH4	1.4 × 10^−7^	216	0.04 (measured)
NH3	6.8 × 10^−8^	439.5	0.1
CO2	1.6 × 10^−9^	17697.5	1.56
HF	1.2 × 10^−6^	24.6	0.0038
H2O	3.5 × 10^−7^	84.7	0.014
